# Enhancement of Harbin ice and snow tourism destination competitiveness: A large-scale data study based on sentiment analysis and Latent Dirichlet Allocation

**DOI:** 10.1371/journal.pone.0319435

**Published:** 2025-03-21

**Authors:** Bin Jiang, Chunxiang Zhang, Yingyin Cui, JiuLian Zhu, Zhennan Liu

**Affiliations:** 1 College of Science, Shihezi University, Shihezi, People’s Republic of China; 2 College of Marxism, Dali University, Dali, People’s Republic of China; Zhejiang Gongshang University, CHINA

## Abstract

In recent years, the ice and snow tourism industry has exhibited a concurrent state of vigorous expansion and intense rivalry. Strengthening competitiveness is of paramount significance for attaining a dominant position in the market. Thus, this study endeavors to dissect the multi-dimensional determinants of the competitiveness of ice and snow tourism destinations from the demand perspective and establish an evaluation framework. Leveraging 48,420 tourist reviews sourced from ten highly reputed ice and snow tourist attractions in Harbin, text features were extracted via the application of Term Frequency - Inverse Document Frequency (TF-IDF). Subsequently, the Latent Dirichlet Allocation (LDA) topic model was deployed to precisely extract key themes. The sentiment inclination was gauged by SnowNLP. Eventually, the Importance - Performance (IPA) model was utilized to analyze the strengths and weaknesses of the ice and snow tourism competitiveness. The outcomes are as follows: (1) Seven prominent themes, namely tourist activities, environment, resources, historical and cultural aspects, cost - performance, service, and experience, were recognized as potent driving forces. (2) Tourists manifested positive emotions and a high degree of approval. (3) The competitiveness score of the Harbin ice and snow tourism destination was determined to be 0.64, with tourist activities constituting the advantage, whereas resources and environment necessitating enhancement. This study transcends the constraints of traditional supply-side-centered research. Innovatively, tourist sentiment was integrated into the evaluation system, thereby augmenting the connotations of the competitiveness evaluation model. Based on the LDA topic clustering results, sentiment analysis was conducted, enhancing the accuracy of portraying tourists’ inner experiences. Concrete and forward-looking strategic recommendations were proffered for augmenting the competitiveness of ice and snow tourism destinations, thereby furnishing theoretical and practical guidance for the high-quality progression of ice and snow tourism.

## 1. Introduction

Ice and snow tourism has emerged as a crucial component within China’s ice and snow economy [1]. Following the successful conclusion of the Beijing Winter Olympics, the ice and snow tourism sector in China has entered a “post-Winter Olympics” era, characterized by diversified and high-quality development patterns [2]. Data from the “China Ice and Snow Tourism Development Report (2024)” released by the China Tourism Research Institute indicates that during 2022–2023, the number of ice and snow tourism trips in China reached 312 million, with corresponding revenues amounting to 349 billion yuan. It is anticipated that in the 2023–2024 ice and snow season, the number of China’s ice and snow leisure travels will exceed 400 million, and the projected ice and snow recreation tourism revenue is expected to reach 550 billion yuan [3]. Therefore, the booming ice and snow tourism not only drives the development of related industries but also gradually changes people’s lifestyles and consumption choices, and is gradually evolving into a “new national trend” [4]. The pursuit of high-quality ice and snow tourism depends on the continuous enhancement of destination competitiveness. Destination competitiveness represents a destination’s ability to offer attractive elements such as tourism attractions, services, and supporting facilities to the market [5], thereby obtaining economic and social benefits and gaining a competitive edge [6]. This has been a significant focus of research in the field of tourism geography [7].

Competitiveness was initially studied at the firm level by Porter in the 1980s [8]. However, it was not until 1993 that scholars first identified the four key elements underlying competitive success in the tourism domain, thereby initiating the research on destination competitiveness [9]. Subsequently, scholars have directed their attention to various aspects, including the definition of tourism destination competitiveness [10–12], the identification of influencing factors [13,14], evaluation models [15–17], sustainable development [18], and intelligent tourism [19]. Notably, academic research has predominantly centered on the development of evaluation models for tourism destination competitiveness. As Cronjé and du Plessis (2020) and J. Xu and Au (2023) pointed out, existing models are mainly constructed from the supply-side perspective, emphasizing the roles of the government and key stakeholders [7,20]. For example, Javed and Tučková (2020) developed a model based on the Tourism Area Life Cycle (TALC) model to explore the role of the government in the competitiveness of tourism destinations and its relationships [21]. In contrast, relatively few models have been developed from the demand-side perspective, which primarily focuses on tourists. For instance, Neto, Dimmock, Lohmann, and Scott (2020) examined the extent to which tourists’ travel experiences affect the importance of destination competitiveness [22]. Even fewer studies have adopted an integrated supply and demand approach. For example, Murayama, Brown, Hallak, and Matsuoka (2022) analyzed the competitiveness of the Hayama district in Japan from both the supply side (local companies) and the demand side (tourists) [23]. Additionally, different types of destinations, such as specific countries or regions [24], islands [25], cities [26], and sports destinations [27], have been explored in the research on destination competitiveness.

Through a comprehensive review of the existing literature, the following has been determined: (1) Although significant progress has been made in the research on tourism destination competitiveness evaluation models, the demand-side perspective remains relatively underdeveloped and requires further exploration. (2) Despite the strong momentum of ice and snow tourism development, there is a lack of in-depth research on the competitiveness of ice and snow tourism destinations. (3) Currently, in the process of constructing an evaluation model from the demand-side, tourists’ opinions are mainly collected through questionnaires and interviews, which demands substantial human resources and is constrained by financial and time limitations. Fortunately, with the rapid advancement of Internet technology, tourists are increasingly inclined to share their travel experiences, observations, and perceptions online [28,29]. Tourists’ review texts can serve as a valuable source of information for analyzing tourism competitiveness. Moreover, the application of text analysis techniques can reduce the costs associated with data collection, enable the analysis of a broader range of samples, and enhance the generalizability of the results [30,31].

In light of the above, the tourists’ review texts obtained from Ctrip and Tongcheng, two prominent Chinese tourism service platforms, are used as the raw data. Initially, Python is employed for text preprocessing. Subsequently, keywords are extracted and the themes of the review texts are clustered using the Term Frequency - Inverse Document Frequency (TF-IDF) algorithm and the Latent Dirichlet Allocation (LDA) method. The TF-IDF algorithm, widely used in text mining and information retrieval, measures the significance of words in a text by considering both their within-text frequency and their rarity across the corpus, facilitating the identification of words that encapsulate the essence of tourists’ reviews. Conversely, the LDA method, a probabilistic topic model, can automatically uncover latent topic structures from extensive text data, enabling the classification of review texts into different themes. Through this process, the tourists’ perceptions during ice and snow tourism participation are analyzed. Thereafter, SnowNLP, a Python-based natural language processing library designed for Chinese text analysis with functions such as word segmentation, part-of-speech tagging, and sentiment analysis, is utilized to evaluate the tourist satisfaction of all texts and corresponding data for each theme. This model quantifies the sentiment of tourists’ reviews within the 0–1 range, where a higher value indicates a more positive sentiment and vice versa. Finally, based on the understanding of ice and snow tourism and tourist satisfaction, a competitiveness evaluation system for ice and snow tourism destinations is constructed, and the Importance-Performance Analysis (IPA) model is established to analyze the strengths and weaknesses of such destinations.

This study distinguishes itself from previous works in several significant respects. Firstly, from a research perspective, it has pioneered a novel approach by deeply analyzing the rich and unstructured online review texts of tourists. Unlike many previous studies that predominantly focused on the supply side or adopted a simplistic view of the demand side, our research comprehensively captures the detailed and diverse perceptions of tourists during their ice and snow tourism experiences. This holistic understanding of tourists’ experiences has been scarcely explored in the research on destination competitiveness, thereby providing a fresh and broader perspective and enriching our knowledge of the factors influencing destination competitiveness. Secondly, in terms of research methodology, we have adopted a unique combination of advanced text analysis techniques and made adaptive modifications. While the TF-IDF and LDA methods are commonly employed in text mining, our application in the specific context of ice and snow tourism is innovative. We have adjusted the TF-IDF algorithm to more effectively account for the special vocabulary and semantic nuances in ice and snow tourism reviews, enabling a more profound exploration of the factors shaping tourists’ perceptions and satisfaction. Finally, in terms of practical application, we offer practical and feasible strategies for the high-quality development of ice and snow tourism destinations. By utilizing the Importance-Performance Analysis (IPA) model, we can identify the strengths and weaknesses of destinations and provide specific and actionable improvement suggestions. Compared with many previous studies that often lingered at the stage of theoretical evaluation, this direct linkage between research results and practical solutions represents a notable advancement, making our research more relevant and valuable to industry stakeholders.

The research objective of this paper is to construct a comprehensive and innovative competitiveness evaluation model for ice and snow tourism destinations based on tourists’ in-depth perceptions. By leveraging a large amount of online review data, we aim to uncover the factors that influence tourists’ satisfaction and loyalty, thereby assisting destination managers and stakeholders in making informed decisions for sustainable development and enhanced competitiveness.

The main research contributions of this paper are as follows:

(1) To develop an advanced text analysis framework that can efficiently process and analyze the complex and unstructured online review texts of ice and snow tourists, extracting valuable insights and key factors related to their tourism experiences.
(2) To establish a robust competitiveness evaluation model that combines the quantitative and qualitative aspects of tourists’ perceptions, with a particular emphasis on the unique characteristics and requirements of ice and snow tourism.
(3) To determine the critical success factors and areas for improvement of ice and snow tourism destinations through the application of the IPA model, and to provide targeted strategies to enhance destination competitiveness.

## 2. Literature review

### 2.1. Tourism perception and its emotional dimensions in tourism destination competitiveness

Tourism perception, which encompasses tourists’ subjective evaluations of the natural environment, cultural milieu, service quality, and facility provisions of a destination during their sojourns, plays a fundamental role in molding the overall tourism experience. Georgiana-Denisse Savin (2021) conducted an exhaustive review of the literature pertaining to the application of eye-tracking technology within the tourism domain, identifying tourists’ visual focal points and behavioral patterns in response to diverse tourism stimuli such as exhibitions and restaurant menus. This work has furnished a novel perspective and empirical underpinning for the exploration of tourism perception from a visual perception vantage point [32]. Tourism perception not only exerts a pronounced influence on tourist satisfaction and loyalty but also functions as a catalyst for word-of-mouth dissemination, thereby augmenting tourist influx [33,34]. Recent investigations have probed deeper into the latent emotional aspects of tourism perception. For instance, Xiao, Fang, Lin, and Chen (2022) undertook an empirical study in Wuyuan, China, with the aim of unraveling the nuanced role of perceived images. Their research demonstrated the profound emotional imprints that destinations leave on tourists and elucidated how these emotions, in turn, sway tourists’ intentions to revisit and recommend the destination [35]. Su, Jia, and Huang (2022) harnessed data from a microblogging platform to construct a model that delineated the emotional repercussions of negative events at tourist destinations, specifically in relation to tourists’ retaliatory and boycott behaviors [36]. Zhengyan Chen (2024) employed natural language processing (NLP) techniques to dissect web text data, ascertaining the average emotional index of traditional villages in Fuzhou, China [37]. This research accentuated the pivotal significance of fostering positive emotional experiences among tourists for the sustainable development of various tourism scenarios. The present study endeavors to further dissect the nexus between the tourism environment and tourists’ emotions within ice and snow tourism destinations, underlining the import of emotional factors across diverse tourism settings. In the context of tourism destination competitiveness research, tourists, as integral stakeholders in the evolution of destination tourism, proffer unique perspectives, perceptions, and values that warrant close attention [38]. Tourism perception, intertwined with emotional experiences, has emerged as a decisive determinant of tourism destination competitiveness [39]. Consequently, this paper undertakes an in-depth analysis of tourists’ comments from a demand-side perspective, with a particular emphasis on the emotional facets of tourism perception, in a bid to unearth the latent factors that impinge on the competitiveness of ice and snow tourism destinations.

### 2.2. Evolution and gaps in tourism destination competitiveness research

Tourism destination competitiveness has long been a central preoccupation in tourism research, given its criticality for destinations to either maintain or expand their market shares [40]. The construction of evaluation models has been a core area of exploration. Pioneering efforts, such as that of Crouch (2021), adopted Porter’s “Diamond Model” as a scaffold to devise a tourism destination competitiveness model, integrating social prosperity, social welfare, and tourism development to appraise the competitiveness of regional tourism destinations [41]. Subsequently, Rheeders and Meyer (2022) advocated the application of a quantitative methodology predicated on partial least squares structural equation modeling (PLS-SEM) to compare the competitiveness of tourism destinations [42]. Moradi (2022) constructed a comprehensive competitiveness evaluation model for sport tourism destinations using the grounded theory approach [43]. S. Yang and Jiang (2022) employed Porter’s diamond model to dissect the competitiveness of the tourism industry in Chinese provinces from 2001 to 2017 [44]. Hai and Yahui (2023) developed a tourism development index evaluation system for world-renowned city tourism destinations, drawing inspiration from the indicator system proposed by the World Economic Forum for gauging the competitiveness of tourism destinations in global economies [45]. However, a critical examination of the extant literature reveals that the majority of evaluation models have been formulated from a supply-side perspective. In contrast, models constructed from a demand-side perspective, especially those that assimilate the emotional and experiential dimensions of tourists, are relatively scarce [7,20]. Ice and snow tourism, as an emerging and distinctive tourism modality, has been witnessing a burgeoning popularity. Notwithstanding, there remains a paucity of comprehensive studies zeroing in on the competitiveness of ice and snow tourism destinations. This study strives to bridge this gap by erecting a competitiveness evaluation system for ice and snow tourism destinations from a demand-side perspective, with a specific focus on integrating the emotional and psychological constituents of tourists’ experiences.

### 2.3. Leveraging tourism text analysis techniques to capture emotional insights

With the popularity of the Internet, the text data generated by travelers on social media platforms and online websites has become a valuable resource in the field of tourism [46,47]. These data are characterized by their large volume and rich content, which not only provide insights into tourists’ genuine feelings and experiences but also contain a wealth of market intelligence and tourism trends [48], thus furnishing invaluable first-hand information for tourism research [49]. Notably, the application of online text data in competitiveness research has been increasing. H. Wang, Gao, Yin, & Liu (2017) employed pattern matching and machine learning techniques to identify comparative relationships in restaurant online reviews, resulting in the construction of a competitiveness analysis model [50]. Y. Liu, Jiang, & Zhao (2019) evaluated the competitive advantage of products from the customer’s perspective by mining user-generated content on social media [51]. Qin, Wang, & Xu (2022) proposed an innovative method to analyze the competitiveness of tourist attractions by leveraging intuitional fuzzy and hesitant fuzzy information obtained from sentiment analysis to rank tourist attractions [52]. Xiaodong Sun (2023) conducted sentiment analysis on online reviews of cruise tourists, identifying the patterns of tourists’ positive and negative emotional expressions triggered by different cruise attributes and elements as well as the differences in market positioning [53]. Ma, Zhu, Cao, & Li (2024) introduced a novel calculation method for competitiveness assessment based on web review data [54]. Nevertheless, despite these advances, there remains a significant scarcity of studies that quantitatively measure the competitiveness of ice and snow tourism by utilizing the emotional insights obtained from sentiment analysis of tourism text review data. In response to this gap, this study combines the Latent Dirichlet Allocation (LDA) technique with SnowNLP sentiment analysis. Firstly, the LDA theme model is used to explore the factors influencing the competitiveness of ice and snow tourism from the tourists’ perspective, with a particular focus on uncovering the emotional and cognitive structures that underpin their experiences. Subsequently, SnowNLP sentiment analysis is utilized to calculate the overall sentiment and the sentiment scores related to each theme, thereby quantifying the emotional valence of tourists’ reviews. Finally, a comprehensive ice and snow tourism destination competitiveness evaluation model is established on this basis, with the emotional dimensions of tourists’ experiences serving as a central tenet.

## 3. Materials and methods

### 3.1. Research area overview

Harbin is geographically located in the southwest of Heilongjiang Province in Northeast China and serves as an important international comprehensive transportation hub on the First Eurasian Continental Bridge and air corridor. The reasons for selecting Harbin as the research area are mainly as follows:Firstly, Harbin is a renowned destination for ice and snow tourism in China, attracting a large number of tourists with its unique ice and snow landscapes. Famous attractions such as the Ice and Snow World, St. Sophia Cathedral, and Central Street have become must-visit places for tourists, thus being highly representative within China. Secondly, Harbin was rated as one of the top ten ice and snow tourism cities in 2024. Thirdly, according to the data from the official website of the Harbin Municipal People’s Government in 2023, during the three-day New Year’s Day holiday, Harbin received a cumulative total of 3.05 million tourists, and the total tourism revenue approached 6 billion yuan, creating a remarkable “miracle” in urban tourism.

Therefore, the analysis of Harbin as an ice and snow tourism destination’s competitiveness can provide valuable references for the high-quality development of ice and snow tourism in China.

### 3.2. Data sources and processing

In the current highly competitive digital tourism market landscape, Ctrip (https://www.ctrip.com), as a leading player in the domestic market, occupies the largest market share among online travel agency (OTA) platforms. OTA, which stands for Online Travel Agency, refers to a platform that enables consumers to book various travel-related services such as hotels, flights, tours, and car rentals through the Internet, integrating multiple travel resources and providing convenient one-stop shopping services for travelers. Meanwhile, Tongcheng Travel (https://www.ly.com), by leveraging the vast WeChat ecosystem, has developed into an OTA platform with a considerable domestic traffic scale and has become a complementary force to Ctrip. Given the prominent advantages and high representativeness demonstrated by the two in terms of tourism market data aggregation, this study selected them as the core data sources, aiming to obtain the most extensive and representative ice and snow tourism-related data, thereby ensuring that the research results can truly and accurately reflect the overall market situation.

The data for this study was mainly collected using the Octopus tool, which is a widely recognized and freely available web crawler software. This tool is equipped with advanced features, enabling researchers to extract the required data from websites without complex coding. The collection period was precisely set from November 1, 2023, to March 1, 2024, which fully considered the seasonal characteristics of ice and snow tourism and also included multiple important holidays and peak tourist periods, such as New Year’s Day and the Spring Festival.

Using the Octopus tool, the comment data of the 10 scenic spots that received high attention in 2023 on the above two platforms (such as the Ice and Snow World, Central Street, Yabuli Ski Resort, and Harbin Polar Park, etc.) were captured, and initially a total of 49,102 pieces of data were collected and shown in Table 1. Given the importance of data accuracy and reliability for the study, the research team adopted a manual deduplication method to clean the data. Firstly, in Excel, the worksheet where the comment data was located was selected, and the “Comment Content” column was used as the screening target column. Through the “Filter” function in the “Data” tab, the screening condition was set to retain only one of the completely identical texts in the “Comment Content” column, thus removing duplicate data. After completing the preliminary screening based on completely identical texts, the team members also conducted a random sampling inspection of the remaining data. By manually reading, they checked whether there were partially duplicate or highly similar texts. For these suspected duplicate texts, a further comparison was made in terms of semantics, key information, and emotional tendency. If it was determined that they had a high degree of repetitiveness and similarity, they were merged or deleted according to the specific situation to ensure the simplicity and independence of the data, while maximizing the retention of the diversity and richness of the data and avoiding the loss of valuable information due to over-screening.

**Table 1:** Data source.

Data source platform	Scenic spots
Ctrip (https://www.ctrip.com) Tongcheng Travel (https://www.ly.com)	1. Ice and Snow World
	2. Northeast Tiger Forest Park
	3. Sun Island Scenic Area
	4. Volga Manor
	5. Polar Park
	6. St. Sophia Cathedral
	7. Central Street
	8. Yabuli Ski Tourism Resort
	9. Songhua River
	10. Sun Island Snow Expo

Subsequently, the research team, relying on the profound professional knowledge and acute judgment accumulated in the field of tourism, conducted an in-depth review of the comments, aiming to identify and remove the system-generated positive comments. Such comments often have obvious characteristics. For example, the text like “User did not comment, system default positive comment” is completely lacking in substantive content, and the text like “Had a great time playing, this scenic spot is worth recommending to friends” has a simple evaluation but is patterned in language, lacks specific details, and has a relatively general emotional expression, unable to truly reflect the personalized experiences and objective evaluations of tourists, and such texts that appear repeatedly. If these system-generated positive comments are mixed into the research data, it is highly likely to seriously mislead the authenticity of the research results, causing deviations in data analysis and further affecting the reliability of the entire research conclusion. After this rigorous and meticulous manual cleaning process, 48,420 pieces of valid data were finally obtained. These refined data laid a solid foundation for the subsequent in-depth analysis and scientific inference of this study, effectively guaranteeing the credibility and validity of the research results.

### 3.3. Research framework

**Fig 1:**
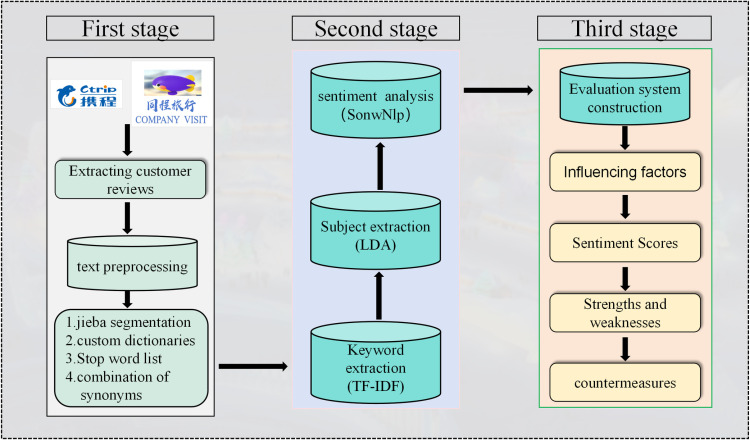
The analytical framework of this study.

The research paradigm of this study consists of three consecutive stages, as shown in Fig 1. In the initial stage, the tourist comments on the target tourist attractions on the Ctrip and Tongcheng Travel platforms were obtained, and then a series of preprocessing procedures were implemented on the comment texts, including using the Jieba word segmentation technology, loading custom dictionaries and stop word lists simultaneously, removing stop words, and merging synonyms to ensure the consistency and relevance of the data.

In the next stage, the mature and widely applied TF-IDF algorithm was used to conduct in-depth mining on the cleaned text data, accurately extracting the representative keywords that can highly summarize the core content and key themes of the text. On this basis, the LDA topic model was introduced to deeply explore the latent topic structures and semantic information hidden behind the massive tourist comment data. Subsequently, the trained SnowNLP model was precisely applied to the comment text analysis under each topic. Through the intelligent calculation and deep semantic understanding ability of the model, the emotional scores of each topic and the overall text were accurately quantified, thus transforming the vague emotional tendencies of tourists towards various aspects of Harbin’s ice and snow tourism into clear, definite, and comparable numerical indicators, realizing the precise quantitative evaluation of the tourist satisfaction level.

In the final stage, the topic clustering results extracted by the LDA topic model were used as the core basis and key dimension to construct a comprehensive, scientific, and systematic evaluation index system for the competitiveness of Harbin’s ice and snow tourism destination. With the help of the IPA model, an intuitive visual chart was presented to deeply evaluate the inherent advantages and disadvantages in the competitiveness of Harbin’s ice and snow tourism destination, providing valuable insights for further analysis and strategic decision-making in the field of ice and snow tourism research.

### 3.4. Research methods

#### 3.4.1. TF-IDF algorithm.

The TF-IDF algorithm is a widely used statistical method in the field of text processing, which is used to evaluate the importance of a word in a text collection or corpus [29]. This algorithm determines the weight of a word by combining the term frequency (TF) (i.e., the number of times a word appears in a single document) with the inverse document frequency (IDF) (i.e., the distribution frequency of the word in the entire document collection). The higher the TF-IDF value, the more important the word is to the document. Therefore, it is commonly used in tasks such as text classification, text summarization, and keyword extraction [55]. The TF-IDF algorithm has the advantages of simplicity, high efficiency, strong interpretability, good robustness, and easy extensibility, and has been widely used and recognized in practical applications. Therefore, this paper uses the TF-IDF algorithm to extract the text keywords of Harbin’s ice and snow tourism comments and calculate the TF-IDF weights, providing a solid and reliable vocabulary foundation for the subsequent text analysis and topic model construction, ensuring that the research can closely focus on the key elements and characteristic highlights of Harbin’s ice and snow tourism, and avoiding unfocused and aimless general analysis.

#### 3.4.2. LDA model.

The LDA (Latent Dirichlet Allocation) is a topic model for text analysis generated based on Bayes’ theorem, which can automatically discover the hidden topic structures from a set of documents [56]. The LDA topic model assumes that each document is composed of several latent topics, and each topic is defined by a group of words through probability distribution [57]. In this way, the LDA topic model can not only decompose a document into a combination of topics but also automatically discover the latent topic structures from a large amount of text data and predict the topic distribution of new documents, providing strong support for text analysis and understanding [58]. The LDA topic model has the characteristics of flexibility and adaptability and can be applied to various natural language processing tasks, and is robust to noise and incompleteness of text data. Therefore, in this paper, the LDA model is used to identify the latent topic information in the tourist comments on Harbin’s ice and snow tourism and obtain the “Document-Topic” and “Topic-Term” distribution matrices. By observing the “Document-Topic” and “Topic-Term” distribution matrices, the consistency is further calculated to determine the optimal number of topics and determine the topic weights. From the macro-topic level, the overall situation and key issues of Harbin’s ice and snow tourism are grasped, laying a solid foundation for further emotional analysis.

#### 3.4.3. SnowNLP sentiment analysis.

SnowNLP is a natural language processing toolbox based on the Python programming language, providing multiple functions, among which sentiment analysis is particularly prominent, and its primary purpose is to simplify the processing and understanding of text data [59]. This tool can judge the sentiment tendency of a given text by applying a sentiment analysis algorithm and generate the corresponding sentiment score. A score close to 1 indicates a positive sentiment, and a score close to 0 indicates a negative sentiment. In addition, SnowNLP allows users to customize the training model, thereby improving the accuracy of sentiment analysis to meet the needs of different application scenarios [60].

Therefore, in this study, SnowNLP was used to conduct sentiment analysis on the comment texts and the topics obtained from LDA. In the initial stage, tourist comments related to similar tourist attractions were collected using the Ctrip and Tongcheng Travel platforms. A total of 13,956 positive comments and 10,177 negative comments were collected and used for model training. The accuracy of the model was measured by the accuracy rate indicator [61]. After calculation, the accuracy rate reached 0.93, thus verifying the availability and reliability of the model. Subsequently, SnowNLP was used to conduct a comprehensive sentiment analysis on the comment texts corresponding to each topic to calculate the sentiment scores, providing key and reliable sentiment data support for the subsequent construction of the competitiveness evaluation system.

#### 3.4.4. Evaluation of the competitiveness of ice and snow tourism destinations.

Firstly, the LDA topic model was used to cluster the comment data, calculate the consistency, determine the optimal number of topics X_n_, and use the clustering results as the evaluation indicators for the competitiveness of the ice and snow tourism destination and determine the topic weights. Secondly, SnowNLP was used to calculate the overall and each topic’s sentiment tendencies and scores, and on this basis, an evaluation system for the competitiveness of the ice and snow tourism destination was constructed. Then, the weighted average method was used to calculate the competitiveness of the ice and snow tourism destination. Moreover, in order to fully consider the overall and each topic’s situations, the average value was taken as the competitiveness value of the ice and snow tourism destination. In this way, the tourists’ emotional experiences were integrated into the quantitative evaluation system of competitiveness, making the evaluation results more comprehensive, reflecting the actual competitiveness level of Harbin’s ice and snow tourism destination in the tourists’ minds, fully considering the tourists’ subjective feelings and market feedback, and providing strong support for the subsequent analysis and decision-making.

The formula is as follows:


$$\text{C}=\frac{{\text{X*S}}_{\text{X}}+\sum_{\text{i}}^{\text{n}}{\text{x}}_{\text{i}}{\text{*s}}_{\text{i}}}{2}$$ (1)

Where: C represents the comprehensive evaluation index of the competitiveness of the ice and snow tourism destination; X_i_ represents the weight of the i-th topic; S_i_ represents the corresponding sentiment score; X represents the overall perception; S_X_ represents the overall sentiment score.

#### 3.4.5. IPA model.

The IPA model is a market regulation marketing analysis model proposed by Martilla and James in 1977, which is used to evaluate the relationship between the importance of a product or service in the customers’ minds and its actual performance [62]. By using importance and performance as the coordinate axes, the model distributes the evaluation indicators into four quadrants, representing the advantage maintenance area, the status quo maintenance area, the low-advantage development area, and the key improvement area, respectively, which helps enterprises to clearly identify the aspects that need to be focused on and improved. This analysis method helps to improve customer satisfaction and provides decision-making references for the marketing strategies of enterprises [63].

The IPA model has become a powerful tool for enterprises to conduct market research and analyze customer satisfaction due to its characteristics of intuitiveness, guidance, flexibility, resource optimization, continuous improvement, and enhanced competitiveness [64]. In the field of tourism, the IPA model has also been widely applied in research on scenic area marketing [65,66], tourism product development [67], destination management [68], and tourist satisfaction analysis [69]. Therefore, this paper constructs an IPA model for the competitiveness of ice and snow tourism destinations. By interpreting the topics falling in different quadrants, the competitive advantages, disadvantages, and potential development opportunities and challenges of Harbin’s ice and snow tourism destination in various aspects are intuitively presented, providing direct, powerful, and visual decision-making reference bases for formulating precise and effective improvement strategies, optimizing resource allocation, and improving tourism products and services in the future.

## 4. Results and analysis

### 4.1. Analysis of tourists’ perceived keywords in Harbin ice and snow tourism

Keywords and thematic vocabularies constitute the fundamental constituents for the efficacious exploration of characteristics, embodying the specific emotional propensities of tourists and manifesting their affective inclinations [70]. By leveraging Python for the extraction of keywords from the web texts of Harbin’s ice and snow tourism, the TF-IDF values of the keywords were computed, and the top 30 pivotal keywords pertaining to the perception of Harbin’s ice and snow tourism were distilled from the text (Table 2).

**Table 2:** Keywords TF-IDF weight value ranking.

Number	Keywords	TF-IDF value	Number	Keywords	TF-IDF value
1	pretty good	0.3524	16	Queuing	0.1047
2	Animals	0.3423	17	A white whale	0.1034
3	Tickets	0.303	18	Place	0.0998
4	Performing	0.2791	19	Central Avenue	0.0986
5	Beautiful	0.2549	20	Special	0.0974
6	Many people	0.239	21	Cost-effective	0.0886
7	Harbin	0.2358	22	Recommended	0.0881
8	Kids	0.1986	23	Songhua River	0.0849
9	Convenience	0.1696	24	Price	0.0844
10	Worth it	0.1685	25	Scenery	0.0818
11	Feeling	0.1485	26	Happy	0.0815
12	Attractions	0.1309	27	Like	0.078
13	Ice and snow world	0.1175	28	Churches	0.0766
14	Ice sculpture	0.1057	29	Polar Pavilion	0.074
15	Architecture	0.1051	30	Funny	0.0736

A higher TF-IDF value intimates a greater depth of tourists’ cognition and a heightened attention to the imagery elements. The scrutiny of the key terms presented in Table 2 reveals that Harbin, Ice and Snow World, Central Street, and Songhua River are accorded relatively elevated rankings, occupying the 7th, 13th, 19th, and 23rd positions respectively, which attests to the fact that tourists evince a preponderant focus on the aforementioned attractions. Correspondingly, the annual influx of tourists received by these attractions is also comparatively substantial, which cogently reflects the image positioning of Harbin’s ice and snow tourism.

**Fig 2:**
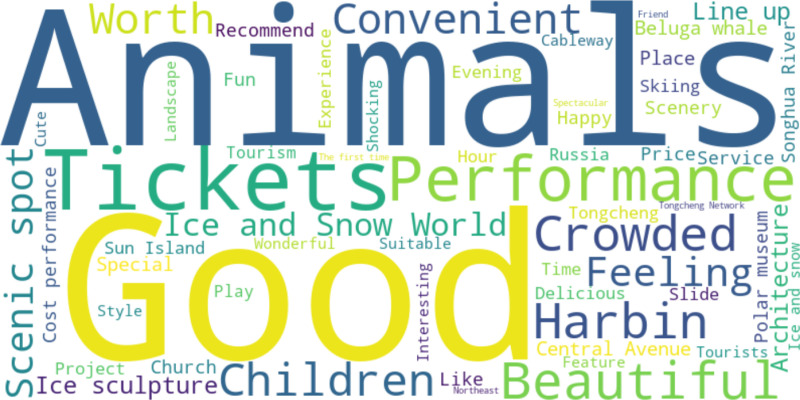
Harbin ice and snow tourism tourists perception word cloud image.

Simultaneously, within the purview of tourism sentiments, positive adjectives such as “nice”, “beautiful”, “convenient”, and “worthwhile” are positioned at relatively prominent ranks, namely the 1st, 5th, 9th, and 10th respectively, signifying that tourists are more content with the tourism evaluation of Harbin and harbor a favorable impression of the tourism experience. Nevertheless, there exist certain pejorative adjectives within the key vocabulary list, such as “bad” and “don’t”, which betoken the extant deficiencies in Harbin’s ice and snow tourism. The emergence of keywords such as “ice sculpture”, “ice and snow”, “skiing”, “slides”, and “ice lanterns” also accentuates the distinctive traits of Harbin’s ice and snow tourism. In the domain of tourism attractions, keywords like “animals”, “performances”, “beluga whales”, and “ice sculptures” are ranked more conspicuously, demonstrating that tourists exhibit an intense interest in experiences intertwined with nature, culture, and entertainment. The manifestation of these high-frequency keywords not only mirrors tourists’ anticipations for unique attractions and activities but also underscores Harbin’s idiosyncratic charm in these respects. The word cloud map visually represents the high-frequency words emblematic of Harbin’s tourism image (Fig 2), and the magnitude of the TF-IDF value within the map is positively correlated with the size of the words.

### 4.2. LDA topic model

**Fig 3:**
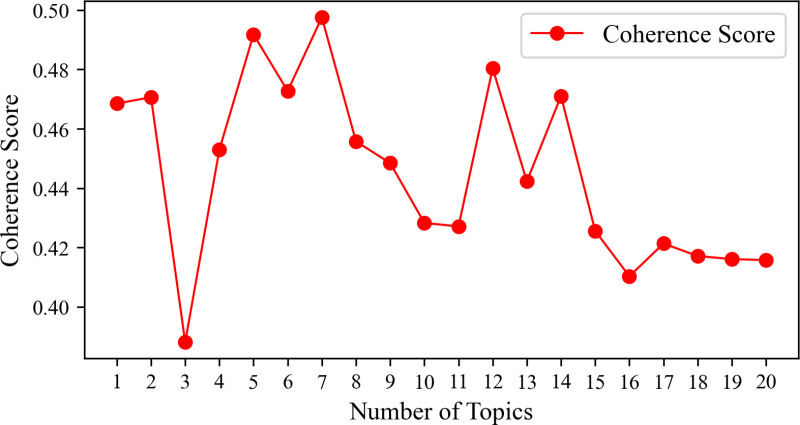
Consistency line chart under different K values.

The LDA topic model is employed to cluster the topics of the preprocessed online reviews of Harbin Ice and Snow Tourism and to extract the associated topics and topic characteristic words. The determination of the optimal number of topics, denoted as K, is requisite for the LDA model to ensure the efficacy of topic extraction. In this research endeavor, the optimal topic count is ascertained through the calculation of coherence, and the adequacy of the topic number is scrutinized using LDAvis. A higher coherence value is indicative of a more favorable clustering effect for the corresponding number of topics. As illustrated in Fig 3, the coherence peaks when the number of themes is set at 7. Consequently, this study opts for seven categories of tourism perceptual imagery themes: tourism activities, tourism environment, ice and snow tourism resources, history and culture, tourism cost-benefit ratio, tourism services, and tourism experience. The outcomes for each category of theme are analyzed as follows (Table 3).

**Table 3:** LDA model topic clustering and sentiment analysis results.

Topic No.	Topic label	Propotion	sentiment score	Top words
Topic 1	Tour Activity	0.238	0.68	Animals, Performing, A white whale, Skiing, Ice sculpture, Slides, A sea elephant, Brilliant, Penguins, Interesting.
Topic 2	Tour Environment	0.194	0.35	Many people, Ropeway, Cold, Queuing, Car, Time, Map, Battery car, Project, Staff.
Topic 3	Ice-snow Tourism Resources	0.172	0.53	Harbin, Songhua River, Sun Island, Park, Attractions, Small town, Central Avenue, Ice and snow world, Polar Pavilion, 5A level.
Topic 4	History and Culture	0.141	0.57	Churches, Architecture Russia, Beautiful, Big, charm, Sophia, History, Style, Features.
Topic 5	Tour Cost-effectiveness	0.11	0.31	Tickets, Convenience, Ctrip, Cost-effective, Price, Online, On the scene, Window, Expensive, Buy.
Topic 6	Tour Services	0.095	0.75	pretty good, Attitude, Service, Worth it, Passionate, Scenery, Place, Scenery, Suitable, Recommended.
Topic 7	Tour Experience	0.05	0.78	Kids, Happy, Good, Likes, Have fun, excellent, Unforgettable, Special, Worthwhile, Not good.

#### 4.2.1. Tour activity.

A profusion of tourism activities constitutes the central allure of the destination (23.8%). Harbin is endowed with a distinctive geographical location and abundant animal resources. Tourists predominantly direct their attention towards elements such as animals, performances, beluga whales, and skiing. In 2023, Harbin endeavored to establish ‘Meet the Polar Regions’ as a prominent symbol of its ice and snow tourism by cultivating animal star ensembles. The original cultural tourism IP of Harbin Polar Park, namely ‘Amoy Penguin’, has visited multiple locations including Sofia Church, Snow Township, and Yabuli; from November 2023 to June 2024, the exposure of ‘Amoy Penguin’ has surpassed 10 billion (https://www.iaiad.com).

#### 4.2.2. Tour environment.

The tourism environment pertains to the natural and humanistic milieus within which tourists engage in travel, manifesting primarily in the natural scenery of attractions, cultural history, transportation convenience, tourism facilities and equipment, as well as the service level (19.4%). This theme principally centers around tourists’ perceptual imagery of the tourist environment, encompassing aspects such as crowds, cableways, cold temperatures, queuing situations, and vehicular conditions. A salutary tourism environment has the capacity to magnetize tourists, augment the tourism experience, and foster the sustainable development of tourism.

#### 4.2.3. Ice-snow tourism resources.

Ice and snow tourism resources are chiefly implicated in tourists’ perceptual imagery of the theme and serve as the bedrock for the development of ice and snow tourism in Harbin (17.2%), relying principally on human resources with natural resources acting in a supplementary capacity. Terms such as Harbin, parks, attractions, Ice World, Central Street, Songhua River, and Sun Island reflect the copiousness of Harbin’s ice and snow tourism resources. The key vocabulary associated with tourist attractions (spots) recurrently references ice sculptures and ice and snow, thereby denoting the singularity of Harbin’s ice and snow tourism resources.

#### 4.2.4. History and culture.

Culture represents the animating spirit of tourism development (14.1%). This theme is predominantly concerned with tourists’ perceptual imagery of culture, inclusive of elements such as churches, architecture, Russian influence, local flavor, and historical heritage. Architecture stands as the historical patrimony of Harbin and a manifestation of diverse cultures. Harbin is a city renowned for its eclectic array of architectural styles from around the world, earning it the appellation of the ‘architectural art gallery in the city’. It amalgamates styles such as Renaissance, Baroque, Byzantine, Russian, and Eclectic, with Central Street serving as the confluence of these architectural masterpieces. Renowned for its inimitable architectural style and robust cultural ambiance, it ranks among the premier commercial thoroughfares in downtown Harbin and is hailed as the ‘Little Paris of the East’.

#### 4.2.5. Tour cost-effectiveness.

A high tourism cost-benefit ratio implies that tourists can derive greater tourism enjoyment and satisfaction within a circumscribed budget, which directly impinges upon tourists’ tourism experience and satisfaction levels (11%). This theme principally revolves around tourists’ perceptual imagery of the cost-benefit aspect of tourism, incorporating elements such as ticket prices, convenience, portable items, value for money, and pricing. Ticket price is a pivotal factor in gauging the cost-effectiveness of tourism. Generally, attractions offering lower ticket prices or preferential policies are more inclined to draw tourists.

#### 4.2.6. Tour services.

Tourism services exert a substantial influence on the perceptual imagery of a tourist destination (9.5%). This theme is chiefly concerned with tourists’ perceptual imagery of tourism services, as exemplified by terms such as agreeable, attitude, service quality, worthwhile, and enthusiasm. These keywords suggest that tourists express satisfaction with the tourism services of Harbin Ice and Snow Tourism, particularly with respect to service quality, the aesthetics of attractions, and overall value.

#### 4.2.7. Tour experience.

The caliber of the tourism experience directly bears upon the satisfaction and loyalty of tourists (5%). This theme is principally correlated with tourists’ overall evaluation and affective responses towards the attractions, encompassing terms such as delighted, fondness, favorable, impressive, enjoyable, memorable, distinctive, juvenile, worthwhile, and unfavorable. The overall evaluation of attractions is capable of swaying tourists’ choices and perceptions. Generally, a favorable overall evaluation will allure more tourists, augment their trust, and enhance their satisfaction. Tourists are prone to disseminate their positive experiences, which will augment the reputation and popularity of the attraction, amplify its commercial value, and concomitantly increase the return rate of tourists.

### 4.3. SnowNLP sentiment analysis

#### 4.3.1. Overall sentiment analysis.

The sentiment analysis of the online commentaries pertaining to the Harbin Ice and Snow Tourist Attractions serves as a means to discern the emotional proclivities of tourists. By leveraging the SnowNLP sentiment analyzer within the Python framework, the sentiment values of the comments are computed, with the resultant output spanning the range of [0, 1]. A higher score within this range is indicative of a more pronounced positive emotional inclination, whereas a lower score corresponds to a relatively more negative disposition [71].

The psychological and emotional experience ladder theory stratifies tourists’ tourism satisfaction into six distinct categories: “highly satisfied” [0.9, 1.0], “moderately satisfied” [0.75, 0.9], “somewhat satisfied” [0.6, 0.75], “average” [0.5, 0.6], “mildly dissatisfied” [0.4, 0.5], and “highly dissatisfied” [0.3, 0.4], as well as “extremely dissatisfied” [0, 0.3]. For the purposes of subsequent analysis, these six categories of tourist satisfaction are further consolidated into three overarching classes: “positive emotions” [0.6, 1.0], “neutral emotions” [0.5, 0.6], and “negative emotions” [0, 0.5] [72].

The distribution of the emotional propensity within the comments regarding Harbin Ice and Snow Tourism is presented in Table 4. Among the 48,420 comments analyzed, positive comments constituted 73.88%, negative comments accounted for 24.23%, those expressing high satisfaction represented 64.13%, and those denoting high dissatisfaction amounted to 20.72%. It is thus evident that, on the whole, tourists’ experiences of Harbin ice and snow tourism exhibit a positive inclination, albeit with a conspicuous bipolar tendency.

**Table 4:** Sentiment analysis table of tourist reviews.

Emotion category	Number of comments	Proportion (%)	Strength	Number of comments	Proportion (%)
Positive (0.6–1)	35642	73.88	Very satisfied (0.9–1)	30934	64.13
			Very satisfied (0.75–0.9)	3059	6.34
			Satisfactory (0.6–0.75)	1649	3.42
Neutral (0.5–0.6)	909	1.88	General (0.5–0.6)	909	1.88
Negative (0–0.5)	11689	24.23	Not satisfied (0.4–0.5)	833	1.73
			Very dissatisfied (0.3–0.4)	862	1.79
			Very dissatisfied (0–0.3)	9994	20.72
Total	48240	100		48240	100

**Fig 4:**
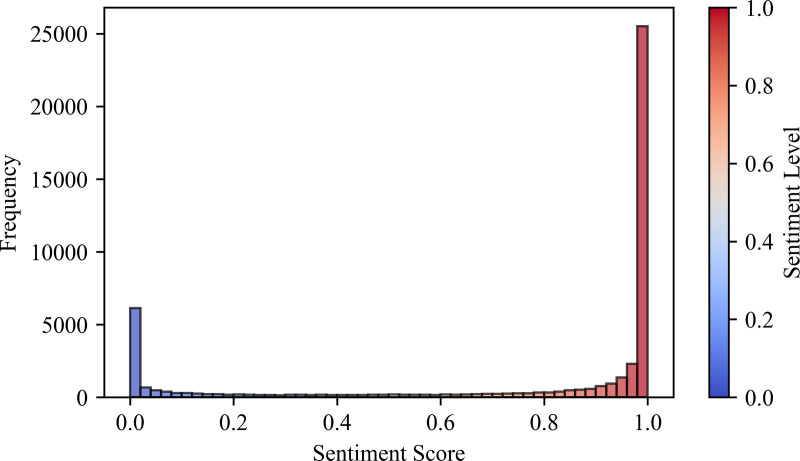
Histogram of emotion score distribution.

Subsequently, as illustrated in Fig 4, the distribution of the emotional propensity of Harbin ice and snow tourism is depicted. The overall mean emotional value of the comments was calculated to be 0.7452, signifying that the general emotional stance of tourists towards Harbin ice and snow tourism is preponderantly positive. The majority of tourists evinced a greater degree of satisfaction with Harbin ice and snow tourism, which is congruent with psychological anticipations. Notably, there was a relatively larger quantity of extremely positive comments with sentiment scores exceeding 0.8, followed by a considerable number of extremely negative comments with scores below 0.2. In contrast, the number of comments falling within the neutral range and those intermediate between the two extreme sentiments was relatively sparse.

#### 4.3.2. Topic sentiment analysis.

Subsequent to the LDA theme clustering process, the SnowNLP library within the Python programming environment was invoked to compute the sentiment values associated with themes and keywords. Each theme’s sentiment score was subsequently substituted with the average of the sentiment scores of the top ten theme words. The resultant data are presented in Table 3, as follows: tourism experience, 0.78; tourism service, 0.75; tourism activities, 0.68; history and culture, 0.57; ice and snow tourism resources, 0.53; tourism environment, 0.35; and tourism cost-effectiveness, 0.31.

Tourists’ positive emotional inclination towards Harbin ice and snow tourism predominantly emanates from the following aspects: (1) Tourism experience, which attained an emotional score of 0.78. The comments in this regard noted, “The seamless integration of ice and electrical elements presents a simply magnificent spectacle. The facilities within the scenic area are impeccable, and the staff members are both hospitable and considerate. The Harbin Ice and Snow World incontrovertibly ranks among the must-visit attractions in one’s lifetime.” Evidently, Harbin ice and snow tourism has met tourists’ psychological anticipations. An analysis of the keywords reveals that the overall perception of tourists towards Harbin is favorable; the experience is gratifying, albeit with a minor “bad” emotional score of 0.07, intimating the existence of certain areas for improvement. (2) Tourism services, with a sentiment score of 0.75. The comments stated, “This was an extraordinary journey; the Ice and Snow World truly lives up to its acclaim. I wholeheartedly endorse Harbin.” This indicates that tourists are satisfied with the attractions in terms of service attitude and quality. (3) Tourism activities, possessing a sentiment score of 0.68. The comments mentioned, “There are ice sculptures, snow sculptures, ice lanterns, snow rings, and ice slides, among other attractions, all of which are available for free enjoyment. It is truly delightful.” In combination with the comments and keywords, it is discernible that Harbin ice and snow tourism entices a substantial number of tourists by virtue of its unique ice and snow culture and diverse tourism activities, with the beluga whale show and the Harbin Ice Show being particularly well-received.

Conversely, tourists’ negative affective propensity towards Harbin ice and snow tourism principally stems from: (1) Tourism cost-effectiveness, which has a sentiment score of 0.31. The comments remarked, “The cost-effectiveness is exceedingly low and does not commensurate with the price.” Owing to the protracted queuing time at the attractions, numerous tourists are precluded from experiencing the relevant projects within the allotted time frame. Coupled with the high ticket prices, tourists perceive a disparity between the price paid and the actual experience, thereby leading to a diminished perception of value for money in tourism. (2) Tourism environment, with a sentiment score of 0.35. The comments lamented, “The entire queuing process occurs in sub-zero temperatures of -20 degrees. Standing stationary without any forward movement for two to three hours is an arduous ordeal. I would not recommend this to others.” The high ticket prices, juxtaposed with the less-than-optimal experience, precipitate the negative emotional inclination associated with the perception of low cost-effectiveness.

### 4.4. Calculation and analysis of Harbin ice and snow tourism competitiveness

The ice and snow tourism competitiveness evaluation system was constructed based on the outcomes of the LDA theme clustering and SnowNLP sentiment analysis (as presented in Table 5). The competitiveness of Harbin’s ice and snow tourism was gauged using the weighted average method, yielding a result of 0.64. This comprehensive score serves to delineate the overall performance of Harbin as an ice and snow tourism destination, both in a general sense and with respect to each individual theme, and is deemed generally reasonable. Harbin has achieved a modicum of success in attracting tourists and proffering rich and diverse experiences and activities. Nevertheless, there remains scope for enhancement, particularly in the amelioration of the tourism environment quality and the augmentation of value for money.

**Table 5:** Harbin ice and snow tourism destination competitiveness evaluation system.

Objective level	Tier 1 indicators	Weighting (%)	Sentiment score (%)	Emotional tendency
ice and snow tourism destination competitiveness	overall perception(X)	100	74	Satisfaction
	Tour Activity(x1)	23.8	68	Satisfied
	Tour Environment(x2)	19.4	35	Very dissatisfied
	Ice-snow Tourism Resources(x3)	17.2	53	Average
	History and Culture(x4)	14.1	57	Average
	Tour Cost-effectiveness(x5)	11	31	Very dissatisfied
	Tour Services(x6)	9.5	75	Very satisfied
	Tour Experience(x7)	5	78	Very satisfied

**Fig 5:**
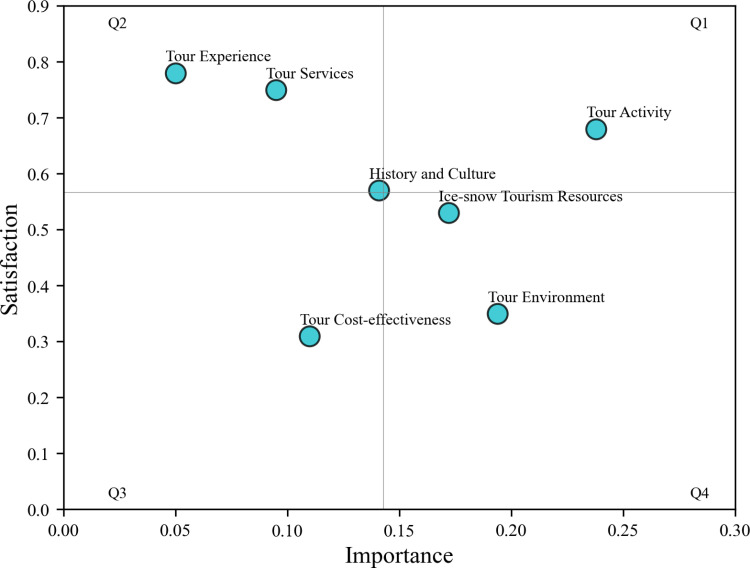
IPA model distribution map.

This study takes the perception and satisfaction of Harbin ice and snow tourism as its foundation. Utilizing the weights of X_1_-X_7_ indicators and the mean value of sentiment scores presented in Table 5 as the demarcation criteria, a four-quadrant diagram of the competitiveness of ice and snow tourism destinations is constructed, and an analysis of the strengths and weaknesses of Harbin’s ice and snow tourism competitiveness is conducted. The results are illustrated in Fig 5. The detailed analyses are expounded as follows:

Among the evaluated attributes, tourism activities fall within the first quadrant, characterized by high importance and high satisfaction, constituting an advantageous aspect. This indicates that the ice and snow tourism activities in Harbin are diverse and congruent with tourists’ psychological anticipations, warranting their preservation and further enhancement.

The attributes situated in the second quadrant encompass tourism experience, tourism services, as well as history and culture. Although these attributes possess a relatively lower degree of criticality, they exhibit a comparatively elevated level of perceived satisfaction. Consequently, there is no immediate exigency to augment investment in these domains, and the existing status quo can be maintained.

Regarding the attributes positioned in the third quadrant, namely tourism cost-effectiveness, it proffers a referential orientation for the amelioration of the competitiveness of Harbin’s ice and snow tourism destination. The current level of satisfaction with tourism cost-effectiveness is relatively meager, thus necessitating continuous scrutiny to detect potential issues and implement practical improvements with adequate resources to augment the competitiveness of ice and snow tourism.

The attributes residing in the fourth quadrant comprise the tourism environment and ice and snow tourism resources. Tourists ascribe a high level of perceived importance to these attributes; however, the corresponding satisfaction levels are deficient. Hence, targeted ameliorative measures should be implemented to bolster the competitiveness of ice and snow tourism destinations.

## 5. Discussion

The exploration of tourism competitiveness holds the potential to confer a competitive advantage upon a destination within a cutthroat market [73]. However, the conceptualization and quantification of tourism destination competitiveness have been frequently regarded as arduous tasks [25,60]. The idiosyncratic viewpoints, cognitions, and appraisals of tourists, who constitute the pivotal stakeholders in the evolution of destination tourism, play a decisive role in ascertaining the competitive standing of a destination [38]. Tourists’ perceptual constructs directly impinge upon the market performance and allure of a destination [39,74]. Accordingly, predicated upon LDA for theme clustering and SnowNLP for sentiment analysis of the review texts pertaining to Harbin’s ice and snow tourism, this paper undertakes an analysis of the themes as perceived by tourists and their emotional proclivities. It formulates a competitiveness evaluation paradigm for ice and snow tourism from the demand vantage point and employs the IPA model to delve into the favorable and unfavorable factors governing the development of Harbin’s ice and snow tourism. Subsequently, a practicable developmental blueprint is proposed with the aim of augmenting the competitiveness of ice and snow tourism.

(1) With regard to the identification of factors that impact the competitiveness of ice and snow tourism, this study employs the LDA model to dissect the relevant factors, which principally encompass tourism activities, the tourism environment, ice and snow tourism resources, historical and cultural elements, the cost-effectiveness ratio of tourism, tourism services, and the overall tourism experience. In alignment with prior investigations [12,22,56,75,76], which have also explored the influencing factors in the field of tourism or destination competitiveness, it is emphasized that a multiplicity of factors, indicators, or dimensions exert an influence on tourism or destination competitiveness. Notably, the significance and satisfaction levels associated with specific indicators are subject to variation contingent upon the distinct types of destinations. In contrast to extant tourism competitiveness studies, the extraction of valuable information from extensive review data via unsupervised learning techniques affords a more objective capture of travelers’ perceptions and anticipations of a destination. This approach is characterized by a broader and deeper scope of data acquisition, enhanced real-time capabilities and cost-effectiveness, as well as more comprehensive analytical capacities.
(2) With regard to the examination of tourists’ sentimental propensity within the context of Harbin ice and snow tourism, this study employs the SnowNLP sentiment analysis technique to compute the sentiment scores associated with tourists’ comments on Harbin’s ice and snow tourism offerings. The overall sentiment exhibits a positive inclination; nevertheless, a bipolar characteristic is conspicuous, and the sentiment distribution is in accordance with the findings of prior investigations [60,77]. The SnowNLP sentiment analysis serves as a valuable tool for discerning tourists’ emotional reactions and stances towards ice and snow tourism destinations, thereby facilitating an in-depth exploration of the subjective experiences. When integrated with the LDA model, the sentimental propensity of each thematic element can be quantified, enabling an objective evaluation of its contribution to the overall competitiveness.
(3) In the establishment of the ice and snow tourism competitiveness evaluation framework, a sequence of research findings have been attained via the employment of review data and sentiment analysis for the quantification of competitiveness [50–52,54]. In this manuscript, we utilize the clustering outcomes of the LDA topic model and the SnowNLP sentiment analysis as the groundwork for devising a competitiveness evaluation model. This approach is capable of furnishing a more exhaustive comprehension of tourists’ remarks and feedback, and of discerning the pivotal elements that impact the tourism experience and destination competitiveness in contrast to the supply-oriented competitiveness evaluation system. Moreover, this model represents a more data-centric and objective paradigm that can offer more profound insights and guidance than the demand-side competitiveness evaluation system constructed through questionnaire surveys and interviews.

Theoretically, it proffers a novel, more scientific and systematic methodology for the assessment of the competitiveness within the domain of ice and snow tourism. Practically, the evaluation of the competitiveness of Harbin’s ice and snow tourism has the capacity to disclose the market status and the level of attractiveness of Harbin’s ice and snow tourism offerings. It can also pinpoint the advantageous and disadvantageous aspects of Harbin’s ice and snow tourism, thereby guiding stakeholders in formulating efficacious development strategies and probing into the pathway for the high-quality progression of ice and snow tourism.

However, as an incipient exploration in the evaluation of ice and snow tourism competitiveness, this paper is not without certain limitations. Primarily, the quantity of the selected research data is relatively scant, given that only the online reviews of ice and snow tourism in Harbin have been examined. This narrow scope fails to holistically represent the perceptions and emotions of tourists engaging in ice and snow tourism across the nation. Future research endeavors could undertake a nationwide horizontal analysis to contrast the disparities in ice and snow tourism competitiveness among different regions. In the realm of sentiment analysis, notwithstanding the employment of the SnowNLP classification method predicated on the Bayesian model, the semantic expressions in Chinese are characterized by variability, and complex sentence structures may on occasion precipitate misjudgments. Hence, for the purposes of augmenting the accuracy of sentiment classification, the integration of sentiment dictionaries into SnowNLP ought to be contemplated in forthcoming research.

## 6. Conclusions and policy recommendations

### 6.1. Conclusions

This study aimed to analyze the competitiveness of Harbin ice and snow tourism through online reviews. The analysis yielded several significant findings. Firstly, it was determined that seven key themes, namely tourism activities, tourism environment, ice and snow tourism resources, history and culture, tourism cost-effectiveness, tourism services, and tourism experience, play a crucial role in shaping the competitiveness of ice and snow tourism. These themes are not only integral components but also interact with each other to influence the overall attractiveness and viability of the tourism destination. Secondly, the sentiment analysis of tourists towards Harbin ice and snow tourism revealed an overall positive perception. However, a more detailed breakdown of sentiment across each theme showed that while tourism experience, services, and activities received favorable sentiment, the sentiment towards tourism scenic spots and architectural style was neutral, and concerns were raised regarding the cost-effectiveness and the environment, which exhibited negative sentiment. This indicates that while certain aspects of the tourism offering are well-received, there are areas that require attention and improvement to enhance the overall tourist experience. Finally, by integrating the LDA model and SnowNLP sentiment analysis, an ice and snow tourism competitiveness evaluation system was developed. The calculated competitiveness score for Harbin’s ice and snow tourism was 0.64. Utilizing the IPA model for further analysis, it was identified that tourism activities represent an advantageous attribute, highlighting the importance of maintaining and enhancing these offerings. In contrast, ice and snow tourism resources and the environment were found to be in urgent need of improvement, and the tourism price-performance ratio was among the sub-optimal attributes that require attention.

### 6.2. Policy recommendations

In light of these conclusions, several policy recommendations are proposed to enhance the competitiveness of Harbin’s ice and snow tourism.

#### 6.2.1. Prioritizing infrastructure development.

Infrastructure development stands as a linchpin in tourism development planning. With the soaring popularity of Harbin’s ice-and-snow tourism, the number of tourists surges exponentially during peak seasons, placing an overwhelming burden on urban transportation and the carrying capacity of scenic areas. Traffic congestion substantially elongates tourists’ travel time, while overcrowding in scenic areas severely diminishes the visiting experience and comfort level.

To address these issues, the following measures can be adopted. Firstly, flexibly regulate the operating hours of popular scenic spots. Based on historical and real - time passenger flow data, extend the opening hours appropriately during tourist peaks to guide tourists to visit at off - peak times, alleviating the passenger flow pressure and enabling tourists to enjoy the scenery at ease. Secondly, vigorously construct an intelligent passenger flow monitoring and management system. Employ Internet of Things, big data, and artificial intelligence technologies to deploy devices at key points in scenic areas. These devices can precisely collect and analyze tourists’ flow, distribution, and movement trajectories in real-time. Through intelligent algorithms, predict passenger flow trends and formulate evacuation plans in advance to prevent overcrowding, ensuring the safety and order of tourists’ visits. Thirdly, actively organize volunteers to participate in traffic guidance and service. Arrange professionally trained volunteers at transportation hubs such as airports and railway stations, as well as on major roads around scenic areas. These volunteers can assist traffic police in maintaining order, provide travel guidance and transfer assistance to tourists, thus relieving traffic congestion, creating a friendly atmosphere, and enhancing tourists’ favorable impression of the city.

#### 6.2.2. Focusing on enriching tourism product and service offerings.

Enriching product offerings and enhancing service quality lie at the core of meeting tourists’ diverse demands and strengthening the attractiveness and competitiveness of the tourist destination. Harbin should closely align with the changing market demands, deeply tap the potential of ice-and-snow tourism resources, and promote the integration of ice-and-snow tourism with various fields such as culture, sports, education, and technology to create a tourism product system with distinct features and high-added-value.

On one hand, continuously diversify the categories of tourism products. While retaining traditional ice-and-snow sightseeing and sports projects, develop innovative products. For instance, launch in - depth ice-and-snow cultural experience tours, allowing tourists to participate in ice carving and ice lantern making, and gain a profound understanding of the local ice-and-snow culture. Conduct ice-and-snow themed research trips, design courses tailored to different age groups of students, and integrate knowledge and culture into practical teaching. Organize international ice-and-snow auto rallies, ice-and-snow marathons, and other events to attract professional athletes and sports enthusiasts, thereby enhancing the international visibility of Harbin’s ice-and-snow tourism.

On the other hand, attach great importance to the improvement of tourism service quality. Establish a one - stop tourism service platform, integrating online and offline resources to facilitate tourists’ itinerary planning, hotel reservation, ticket purchase, and tour guide booking. Strengthen the multilingual training of service staff to ensure smooth communication when receiving international tourists. Increase investment in scenic area infrastructure, improve rest, sanitation, and catering facilities, and reinforce safety guarantees for ice-and-snow sports. Enhance tourists’ experience from the details, cultivate tourists’ loyalty, and generate a favorable word-of-mouth.

#### 6.2.3. Comprehensively strengthening market supervision and public opinion management.

A well - regulated market environment and a positive public opinion atmosphere are crucial guarantees for the sustainable development of Harbin’s ice-and-snow tourism. It is imperative to strengthen market supervision and enhance public opinion management capabilities to create a favorable environment for the healthy development of the industry.

In terms of market supervision, market regulatory authorities must strictly enforce tourism - related laws, regulations, and price policies, and intensify daily inspections and special rectification efforts. Severely crack down on illegal behaviors such as price gouging, false advertising, and forced consumption. Punish illegal enterprises and practitioners in accordance with the law to safeguard tourists’ legitimate rights and interests. Leverage big data to establish a tourism market price monitoring mechanism, conduct real - time monitoring and analysis of prices, and promptly regulate abnormal fluctuations. Improve the tourism complaint handling mechanism, set up a dedicated hotline and an online platform to ensure that tourists’ complaints can be promptly received, efficiently processed, and effectively responded to.

In terms of public opinion management, culture and tourism departments should form a professional team, utilize advanced software and tools to conduct 24 - hour real - time monitoring of public opinion on social media, online forums, and news media platforms regarding Harbin’s ice-and-snow tourism. Through data analysis and sentiment judgment, promptly grasp the public’s evaluation, concerns, and potential demands. Develop a comprehensive public opinion emergency response plan, formulate response strategies and communication scripts in advance for possible negative public opinion scenarios. Once negative public opinion emerges, activate the plan promptly, release authoritative information in a timely manner, respond to public concerns, and guide the direction of public opinion. Strengthen cooperation with the media, and showcase the highlights, achievements, and positive image of Harbin’s ice-and-snow tourism through positive publicity, thereby enhancing its brand reputation and social influence.

In conclusion, through the implementation of the above - mentioned series of policy measures, it is expected to comprehensively enhance the competitiveness of Harbin’s ice-and-snow tourism, achieving high - quality and sustainable development of the tourism industry. This can not only attract more tourists to experience the unique ice-and-snow charm of Harbin, improve tourists’ satisfaction and revisit rate, but also effectively drive regional economic growth, increase employment opportunities, and promote the coordinated development of related industries, laying a solid foundation for Harbin to build an internationally renowned ice-and-snow tourism destination.
